# Interfacial Modulation of Nickel Tungstate by Polyethylene Glycol Toward Enhanced Electrochemical Energy Storage

**DOI:** 10.3390/polym18131639

**Published:** 2026-07-01

**Authors:** Chaitany Jayprakash Raorane, Seong-Cheol Kim

**Affiliations:** School of Chemical Engineering, Yeungnam University, Gyeongsan 38541, Gyeongsanbuk-Do, Republic of Korea

**Keywords:** nickel tungstate, polyethylene glycol, hydrothermal synthesis, asymmetric supercapacitor, interfacial modulation, energy storage

## Abstract

Tailoring electrochemically favorable architectures through polymer-assisted growth regulation offers an effective route for overcoming the structural limitations that restrict the practical performance of pseudocapacitive materials. In this study, a polyethylene glycol (PEG)-mediated interfacial modulation strategy was developed to regulate the structural evolution and electrochemical behavior of hydrothermally synthesized nickel tungstate (NiWO_4_) for asymmetric supercapacitor applications. The influence of PEG concentration (0.1, 0.3, and 0.5 wt%) on crystal growth, morphology evolution, and charge-storage characteristics was systematically investigated. Structural analysis confirmed the successful formation of phase-pure monoclinic NiWO_4_ without detectable impurities, while morphological studies revealed a pronounced PEG-dependent transformation in surface architecture. Among all synthesized electrodes, the optimized NiWO-P3 sample exhibited a highly interconnected porous nanograin framework with improved structural homogeneity and abundant electrochemically accessible interfaces. This favorable morphology significantly facilitated electrolyte penetration, accelerated ion transport, and enhanced redox utilization. Consequently, NiWO-P3 delivered a superior areal capacitance of 9.284 F/cm^2^ at 10 mA/cm^2^ and retained nearly 84% capacitance at elevated current density, demonstrating excellent rate capability. The optimized electrode further exhibited enhanced diffusion kinetics, achieving anodic and cathodic diffusion coefficients of 21.26 × 10^−7^ and 10.55 × 10^−7^ cm^2^/s, respectively, along with remarkable cycling durability of 85.12% after 12,000 cycles. Furthermore, the fabricated NiWO-P3//AC asymmetric supercapacitor demonstrated (ASD) promising electrochemical reversibility and prolonged operational stability, highlighting PEG-assisted interfacial engineering as an effective strategy for advancing high-performance tungstate-based energy-storage materials.

## 1. Introduction

The electrochemical performance of energy-storage materials is governed not only by their intrinsic chemical composition but also by how efficiently their structural architecture facilitates ion and electron transport during charge-storage processes [1,2,3]. In pseudocapacitive systems, even materials possessing abundant redox-active centers frequently fail to achieve their full electrochemical potential because of restricted ion diffusion, insufficient electrolyte accessibility, and sluggish charge transfer kinetics [4,5,6]. Consequently, recent developments in supercapacitor research have increasingly shifted toward structure-regulated material engineering, where morphology optimization and controlled growth strategies are employed to maximize electrochemical utilization and long-term stability [7,8].

Supercapacitors have emerged as attractive electrochemical energy-storage devices owing to their high-power density, rapid charge–discharge capability, and exceptional cycling durability [9,10]. Nevertheless, their relatively lower energy density compared with battery systems continues to limit broader technological implementation [11]. Since the electrochemical performance of supercapacitors is largely dictated by electrode materials, extensive research efforts have focused on developing advanced pseudocapacitive materials capable of delivering rapid charge transport while maintaining structural robustness during repeated cycling [12]. In this regard, transition metal oxides have received significant attention because of their rich oxidation states and fast reversible Faradaic reactions, which enable substantially higher capacitance than conventional carbon-based materials [13,14].

Among various transition metal oxides, transition metal tungstates have attracted increasing interest due to their favorable electrochemical activity, structural robustness, and tunable electronic properties [15]. Several tungstate systems, including cobalt tungstate (CoWO_4_), manganese tungstate (MnWO_4_), zinc tungstate (ZnWO_4_), and NiWO_4_, have been explored for electrochemical energy storage because of their tunable electronic structures and favorable redox characteristics [16,17]. Among these, NiWO_4_ has emerged as a particularly promising pseudocapacitive material owing to the synergistic contribution of nickel and tungsten species within a stable oxide framework [18]. Nickel provides rich electrochemical activity through reversible valence transitions, whereas tungsten contributes structural integrity and electrochemical durability, making NiWO_4_ highly attractive for high-performance supercapacitor applications [19].

Driven by these advantages, considerable efforts have been devoted to improving the electrochemical characteristics of NiWO_4_ through morphology engineering and compositional modification [20]. For example, hydrothermally synthesized NiWO_4_ microflowers reported by Shembade et al. exhibited a high specific capacitance of 712 F/g with nearly 95% retention after 5000 cycles, while also demonstrating promising electrocatalytic activity toward water splitting [21]. Similarly, Niu et al. developed amorphous NiWO_4_ nanostructures, where the sample synthesized at 70 °C delivered a capacitance of 586.2 F/g, and the assembled asymmetric supercapacitor achieved an energy density of 25.3 Wh kg^−1^ with 91.4% retention after 5000 cycles [22]. In another study, Ikram et al. synthesized chain-like NiWO_4_ nanostructures through a hydrothermal route, achieving a remarkably high specific capacitance of 1524 F/g at 0.5 A/g and an energy density of 32.27 Wh/kg, emphasizing the importance of structural architecture in governing electrochemical performance [23]. Furthermore, Elanthamilan et al. developed a NiWO_4_@PANI nanocomposite through hydrothermal synthesis followed by polymerization, where synergistic interactions between NiWO_4_ and conductive polymer networks resulted in enhanced electrochemical performance in a hybrid supercapacitor system [24].

Despite these encouraging developments, the electrochemical performance of NiWO_4_ remains strongly influenced by its structural organization. Particle agglomeration, limited electroactive surface exposure, and restricted electrolyte penetration frequently suppress ion transport and hinder effective utilization of redox-active sites [25]. These limitations indicate that achieving structurally optimized NiWO_4_ architectures remains essential for improving electrochemical kinetics and maximizing practical charge-storage performance [26]. Among various synthesis approaches, hydrothermal methods provide an effective platform for controlling nucleation and crystal growth under relatively mild reaction conditions, enabling improved morphology and structural uniformity [27,28]. More importantly, the incorporation of suitable structure-directing agents during synthesis can substantially influence particle assembly and microstructural evolution, making polymer-assisted growth regulation an attractive strategy for optimizing electrochemically active materials [29]. In this regard, polyethylene glycol (PEG), a widely utilized polymeric additive, has demonstrated considerable potential for regulating crystal growth, suppressing particle agglomeration, and directing morphology evolution during synthesis. Since electrochemical behavior is highly dependent on structural organization, PEG-assisted growth regulation may provide an effective route for improving electroactive accessibility and ion transport in NiWO_4_ systems. However, the influence of PEG remains highly concentration dependent, and systematic understanding of PEG-mediated structural evolution in hydrothermally synthesized NiWO_4_ remains limited.

Although substantial progress has been achieved in NiWO_4_-based electrodes, most reported studies have primarily focused on compositional tuning, hybrid structures, or conductive material integration. Comparatively less attention has been devoted to understanding how controlled PEG incorporation influences morphology evolution and electrochemical behavior in NiWO_4_ systems. Establishing this relationship is therefore important for advancing rational structure engineering of tungstate-based electrodes for high-performance energy-storage applications. Furthermore, asymmetric supercapacitors have attracted increasing attention because of their ability to improve energy density through expanded operating voltage windows and complementary charge-storage mechanisms, thereby emphasizing the need for structurally optimized pseudocapacitive electrodes with enhanced electrochemical accessibility.

Motivated by these considerations, the present study explores a polymer-assisted strategy for tailoring the electrochemical architecture of NiWO_4_ through controlled incorporation of polyethylene glycol during hydrothermal synthesis. By systematically varying the PEG concentration (0.1, 0.3, and 0.5 wt%), this work aims to understand its role in directing morphology evolution and regulating the electrochemical behavior of NiWO_4_ for asymmetric supercapacitor applications. Particular emphasis is placed on establishing the relationship between PEG-mediated structural modulation and charge-storage performance to identify an effective pathway for improving ion accessibility, electrochemical kinetics, and overall energy-storage capability. The findings of this study are expected to provide valuable insight into polymer-assisted engineering of tungstate-based materials for next-generation high-performance asymmetric supercapacitors.

## 2. Experimental Section

### 2.1. Materials

Nickel nitrate hexahydrate (Ni(NO_3_)_2_·6H_2_O, Sigma-Aldrich, St. Louis, MO, USA), sodium tungstate dihydrate (Na_2_WO_4_·2H_2_O, Sigma-Aldrich, U.S.), polyethylene glycol (PEG, average molecular weight (A.M.W.)—20,000 g mol^−1^, Sigma-Aldrich, U.S.), oxalic acid (H_2_C_2_O_4_, Sigma-Aldrich, U.S.), sodium hydroxide (NaOH), hydrochloric acid (HCl), ethanol, and deionized (DI) water were used as received without further purification. Commercial nickel foam (NF) was used as the conductive substrate for the growth of NiWO_4_ electrodes.

### 2.2. Synthesis of PEG-Assisted NiWO_4_ Electrodes

**Figure 1:**
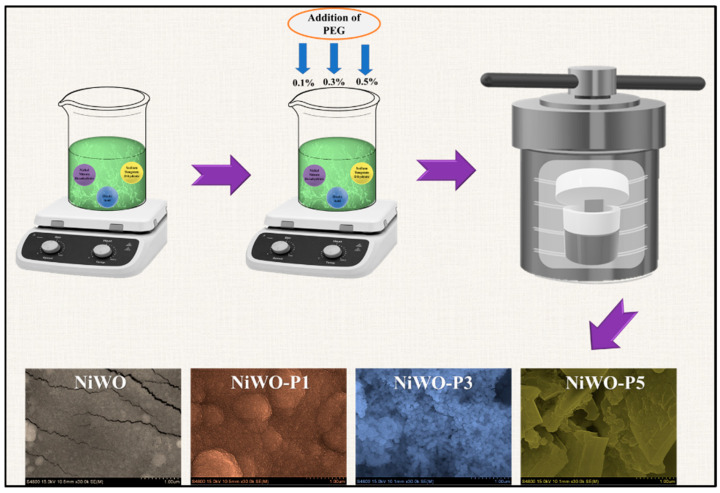
Schematic illustration of the PEG-assisted hydrothermal synthesis process for NiWO, NiWO-P1, NiWO-P3, and NiWO-P5 electrodes.

PEG-assisted NiWO_4_ electrodes were synthesized directly on nickel foam through a hydrothermal method using PEG as a polymeric growth regulator. In a typical synthesis, 0.05 M Ni(NO_3_)_2_·6H_2_O and 0.05 M Na_2_WO_4_·2H_2_O were dissolved separately in 100 mL of DI water under continuous magnetic stirring until homogeneous solutions were obtained. Subsequently, the Na_2_WO_4_·2H_2_O solution was slowly added to the Ni(NO_3_)_2_·6H_2_O solution under constant stirring to form a uniform precursor solution. Thereafter, the required amount of H_2_C_2_O_4_ corresponding to a concentration of 0.01 M was introduced into the mixed solution and stirred for 20 min to facilitate controlled nucleation during the hydrothermal process. The pH of the resulting solution was adjusted to 8 using 1 M NaOH solution. PEG was subsequently incorporated into the precursor solution at concentrations of 0.1, 0.3, and 0.5 wt% (with respect to the total weight of the reaction solution), followed by continuous stirring for an additional 30 min to ensure homogeneous dispersion throughout the reaction medium. Prior to hydrothermal treatment, nickel foam (2 × 3 cm^2^) was ultrasonically cleaned in 3 M HCl, ethanol, and DI water for 10 min each to remove surface impurities and oxide layers. The cleaned nickel foam substrates were then immersed in the prepared precursor solution and transferred into a 100 mL Teflon-lined stainless-steel autoclave**.** The hydrothermal reaction was conducted at 180 °C for 12 h. After naturally cooling to room temperature, the obtained electrodes were removed, thoroughly washed several times with DI water and ethanol to eliminate residual impurities, and dried at 80 °C overnight. The dried samples were subsequently calcined in air at 500 °C for 2 h to improve crystallinity and structural stability. For comparison, the pristine sample synthesized without PEG was designated as NiWO, whereas the PEG-assisted samples prepared with 0.1, 0.3, and 0.5 wt% PEG were denoted as NiWO-P1, NiWO-P3, and NiWO-P5, respectively. A schematic illustration of the PEG-assisted hydrothermal synthesis process of NiWO_4_ is presented in Figure 1.

### 2.3. Characterization and Electrochemical Evaluation

To verify the successful formation of the synthesized materials and understand the influence of PEG-assisted growth, a series of structural, morphological, and electrochemical analyses were carried out for the NiWO, NiWO-P1, NiWO-P3, and NiWO-P5 electrodes. The crystalline phase and structural purity of the prepared samples were identified using X-ray diffraction (XRD) with Cu Kα radiation (λ = 1.5406 Å). Surface architectural changes arising from different PEG concentrations were examined through field-emission scanning electron microscopy (FESEM), while the elemental composition and elemental dispersion were determined using energy-dispersive X-ray spectroscopy (EDS) and elemental mapping analysis. In addition, the surface chemical states and electronic environment of the optimized electrode were investigated using X-ray photoelectron spectroscopy (XPS). The electrochemical response of the prepared electrodes was assessed using a BioLogic electrochemical workstation employing a three-electrode assembly in 2 M KOH electrolyte. The as-prepared NiWO_4_ deposited NF was directly utilized as the working electrode (1 × 1 cm^2^), whereas Ag/AgCl and Pt wire functioned as the reference and counter electrodes, respectively. To systematically examine the electrochemical behavior, cyclic voltammetry (CV), galvanostatic charge–discharge (GCD), and electrochemical impedance spectroscopy (EIS) measurements were performed to probe the capacitive characteristics, charge-transfer behavior, and ion transport kinetics of the synthesized electrodes. All electrochemical investigations were performed at room temperature.

## 3. Results and Discussion

### 3.1. XRD Elucidation

**Figure 2:**
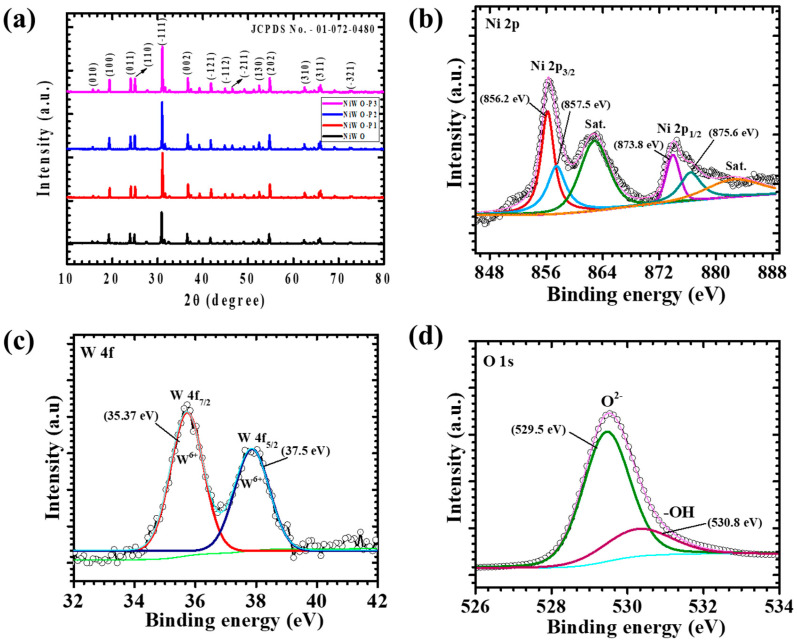
(**a**) XRD patterns of NiWO, NiWO-P1, NiWO-P3, and NiWO-P5 electrodes. High-resolution XPS spectra of the optimized NiWO-P3 electrode: (**b**) Ni 2p, (**c**) W 4f, and (**d**) O 1s.

The crystalline structure and phase composition of the synthesized NiWO, NiWO-P1, NiWO-P3, and NiWO-P5 electrodes were examined by XRD, and the obtained patterns are shown in Figure 2a. All samples display distinct and well-resolved diffraction peaks, confirming the successful crystallization of NiWO_4_ through the hydrothermal route [30]. No additional diffraction peaks associated with secondary phases or impurities are detected, indicating the formation of phase-pure NiWO_4_ irrespective of PEG incorporation. The major diffraction peaks located at approximately 15.6°, 19.4°, 24.1°, 24.8°, 30.5°, 31.2°, 36.3°, 40.2°, 41.74°, 44.7°, 46.4°, 49.1°, 52.3°, 54.7°, 62.3°, 65.8° and 72.7° can be indexed to the (010), (100), (011), (110), (−111), (002), (−121), (−112), (−211), (130), (202), (310), (311), and (−321) planes of monoclinic NiWO_4_, in good agreement with the standard JCPDS card no. 01-072-0480. The close agreement between the experimental and standard diffraction patterns verifies the successful formation of the monoclinic crystal phase of nickel tungstate. Although the overall crystal structure remains unchanged after PEG incorporation, slight differences in diffraction intensity and peak definition are evident among the samples, indicating the influence of PEG on crystal growth during synthesis. Compared with pristine NiWO, the PEG-assisted electrodes exhibit relatively sharper and more intense diffraction peaks, suggesting improved crystallinity and better structural ordering. Notably, the NiWO-P3 sample exhibits comparatively stronger peak intensity and improved peak resolution, implying that 0.3 wt% PEG provides favorable conditions for controlled nucleation and crystal growth. In contrast, a slight decrease in peak sharpness is observed for NiWO-P5, indicating that excessive PEG concentration may influence crystal development and structural ordering. These observations suggest that PEG plays an important role in regulating the crystallization behavior of NiWO_4_, with NiWO-P3 exhibiting comparatively improved structural characteristics among the synthesized samples [31].

### 3.2. XPS Analysis

The chemical states and surface electronic environment of the optimized NiWO-P3 electrode were examined through XPS, and the corresponding high-resolution spectra are presented in Figure 2b–d. The obtained spectra confirm the successful formation of the NiWO_4_ structure and provide insight into the oxidation states of the constituent elements [32]. The high-resolution Ni 2p spectrum (Figure 2b) exhibits characteristic spin–orbit splitting along with noticeable shake-up satellite features, confirming the electronic configuration of nickel in the synthesized material. The Ni 2p_3/2_ region is deconvoluted into two peaks centered at approximately 856.2 and 857.5 eV, while the Ni 2p_1/2_ region consists of peaks located at around 873.8 and 875.6 eV. These binding energies are characteristic of Ni^2+^ species and indicate the presence of slightly different nickel coordination environments arising from metal–oxygen interactions within the NiWO_4_ framework. The appearance of satellite peaks at higher binding energies further supports the predominance of the divalent nickel state [33]. The deconvoluted W 4f spectrum (Figure 2c) displays two prominent peaks located at approximately 35.6 and 37.8 eV, corresponding to W 4f_7/2_ and W 4f_5/2_, respectively. The observed spin–orbit splitting confirms the existence of tungsten in the W^6+^ oxidation state, which is consistent with the chemical state generally reported for crystalline tungstate materials. The absence of additional tungsten-related peaks suggests the formation of a chemically stable tungstate phase. The O 1s spectrum (Figure 2d) can be resolved into multiple oxygen-related components. The dominant peak positioned near 529.7 eV is assigned to lattice oxygen species associated with Ni–O and W–O bonding in the NiWO_4_ lattice. Meanwhile, the component centered around 531.2 eV is attributed to surface hydroxyl groups or defect-related oxygen species, whereas the weak higher binding energy contribution corresponds to adsorbed oxygen-containing species on the surface. The coexistence of these oxygen species may facilitate improved interfacial interaction between the electrode and electrolyte during electrochemical operation [34].

### 3.3. Morphological and Elemental Composition

**Figure 3:**
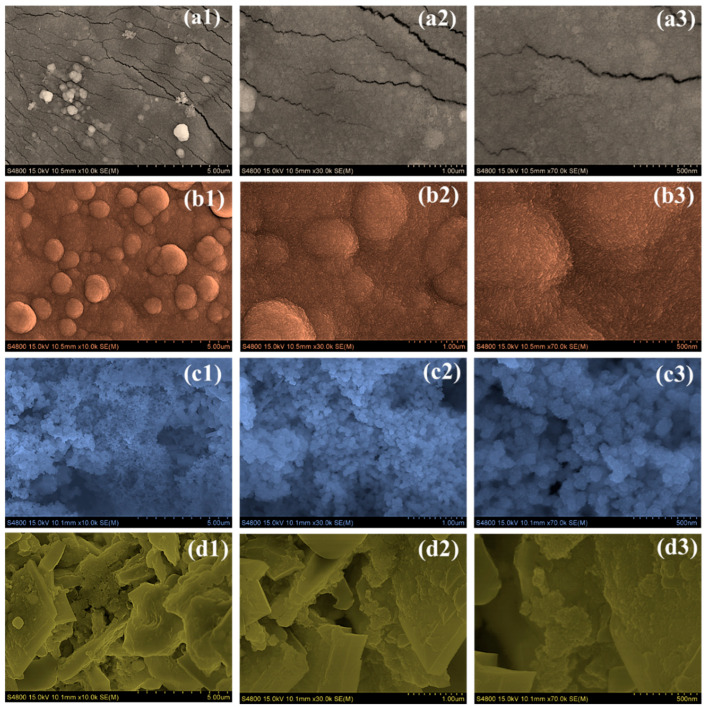
FESEM images of the synthesized electrodes at different magnifications: (**a1**–**a3**) NiWO, (**b1**–**b3**) NiWO-P1, (**c1**–**c3**) NiWO-P3, and (**d1**–**d3**) NiWO-P5.

The morphological features of the synthesized NiWO, NiWO-P1, NiWO-P3, and NiWO-P5 electrodes were examined by FESEM at different magnifications, as presented in Figure 3(a1–d3). The FESEM images reveal a clear evolution in surface architecture with increasing PEG concentration, indicating its important role in regulating the growth and structural organization of NiWO_4_ during hydrothermal synthesis. The pristine NiWO electrode (Figure 3(a1–a3)) exhibits a relatively dense and irregular surface morphology with randomly distributed agglomerated grains. At lower magnification, Figure 3(a1), localized particle clusters are observed along with noticeable crack-like features across the surface. At higher magnifications, Figure 3(a2,a3), the surface appears compact and lacks distinct morphological organization, suggesting uncontrolled crystal growth in the absence of PEG. The compact nature of the surface indicates comparatively limited structural accessibility. Upon introducing 0.1 wt% PEG, a noticeable morphological change is observed for the NiWO-P1 electrode (Figure 3(b1–b3)). The surface becomes populated with relatively uniform microsphere-like structures distributed across the electrode. Compared with pristine NiWO, the morphology appears more organized, indicating that PEG influences the growth behavior during synthesis. However, the microspheres remain closely packed with limited spacing between adjacent structures, resulting in a comparatively compact arrangement. A remarkable morphological refinement is observed for the NiWO-P3 electrode, Figure 3(c1–c3), where the surface transforms into an interconnected assembly of fine nanosized grains. Compared with other samples, NiWO-P3 exhibits a more homogeneous and porous morphology with uniformly distributed nanograins and sufficient interparticle voids. The observed structure suggests that 0.3 wt% PEG provides an optimized growth environment, effectively controlling particle growth while minimizing aggregation. Such an interconnected nanograin network can facilitate improved electrolyte accessibility and more effective electrochemical interaction, which likely contributes to the enhanced electrochemical performance of NiWO-P3. In contrast, increasing the PEG concentration to 0.5 wt% results in another morphological transition in the NiWO-P5 electrode (Figure 3(d1–d3)). The surface becomes dominated by irregular plate-like and stacked flake structures with comparatively larger dimensions. The FESEM images reveal noticeable overlapping and restacking, leading to a less uniform morphology. Although surface roughness is retained, excessive structural growth may reduce the availability of exposed active regions. These observations suggest that PEG concentration strongly influences the structural evolution of NiWO_4_, with NiWO-P3 exhibiting the most favorable morphology among all samples. During hydrothermal synthesis, PEG acts as a structure-directing polymeric regulator that influences nucleation and crystal-growth behavior through weak coordination and surface adsorption interactions with metal ions. The steric stabilization effect of PEG suppresses uncontrolled particle agglomeration and promotes uniform crystallite growth, leading to the formation of interconnected porous nanograin architectures. At the optimized PEG concentration, controlled nucleation and regulated particle assembly facilitate improved structural homogeneity and enhanced electrochemically accessible surface area. However, excessive PEG incorporation may partially inhibit crystallite interconnection and crystal growth due to excessive surface coverage and viscosity-induced diffusion limitation during hydrothermal evolution. Furthermore, PEG-assisted growth also contributes to the generation of interconnected porous channels between assembled nanograins, which are highly beneficial for electrolyte diffusion and rapid ion transport during electrochemical operation [29].

**Figure 4:**
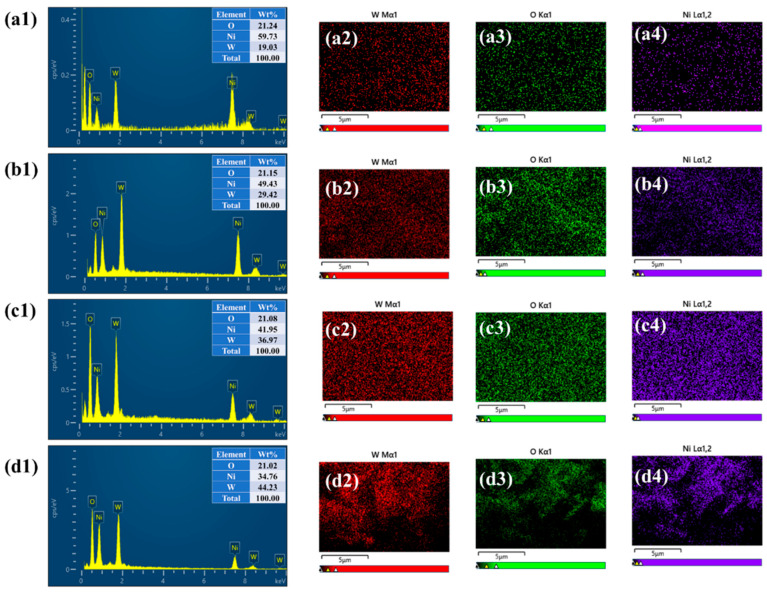
EDS spectra and elemental mapping of the synthesized electrodes: (**a1**–**a4**) NiWO, (**b1**–**b4**) NiWO-P1, (**c1**–**c4**) NiWO-P3, and (**d1**–**d4**) NiWO-P5.

The elemental composition and distribution of the synthesized NiWO, NiWO-P1, NiWO-P3, and NiWO-P5 electrodes were investigated through EDS and elemental mapping analysis, and the corresponding results are presented in Figure 4(a1–d4). The EDS spectra shown in Figure 4(a1–d1) display characteristic signals corresponding to Ni, W, and O, confirming the successful formation of the NiWO_4_ structure in all synthesized samples. No additional impurity-related elemental signals are observed, indicating the compositional purity of the prepared electrodes. The elemental percentages listed in the inset further verify the successful incorporation of nickel, tungsten, and oxygen within the synthesized framework. The slight differences observed in the relative nickel and tungsten elemental percentages among the samples may originate from the PEG-assisted regulation of nucleation and crystal-growth behavior during hydrothermal synthesis. As a polymeric surfactant, PEG influences local ion diffusion, surface adsorption, and crystallite assembly, thereby affecting the surface distribution of constituent elements detected by EDS analysis. Furthermore, the porous and morphology-dependent nature of the electrodes can contribute to localized compositional variation during surface-sensitive EDS measurements. Nevertheless, all samples consistently confirm the presence of Ni, W, and O elements without detectable impurities, validating the successful formation of NiWO_4_ irrespective of PEG incorporation. To further examine the elemental distribution, elemental mapping analysis was carried out for all electrodes. As illustrated in Figure 4(a2–a4,b2–b4,c2–c4,d2–d4), the constituent elements are uniformly distributed throughout the electrode surface. The mapping results reveal homogeneous dispersion of Ni, W, and O without noticeable elemental segregation or localized accumulation, suggesting effective elemental incorporation during synthesis. Among the investigated samples, NiWO-P3 exhibits comparatively more uniform elemental distribution, indicating favorable structural development at the optimized PEG concentration.

## 4. Electrochemical Analysis

**Figure 5:**
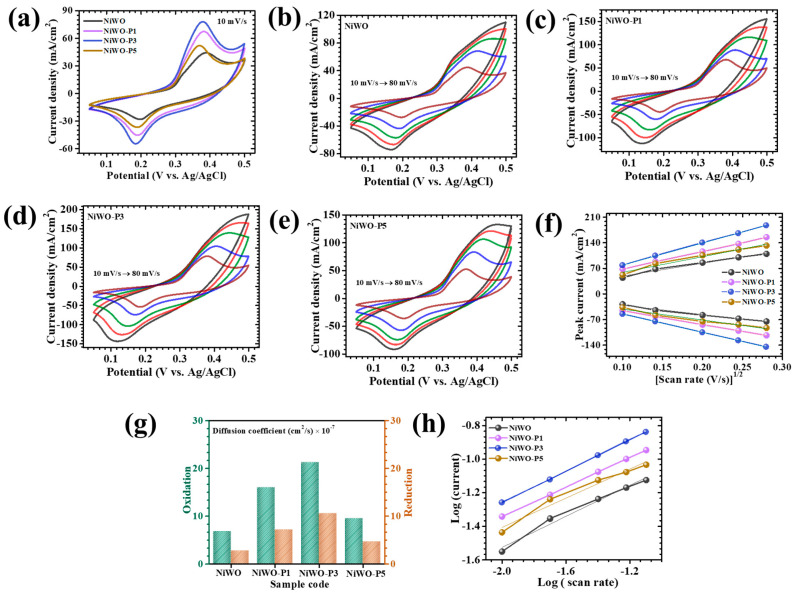
Electrochemical performance of NiWO electrodes in a three-electrode configuration using 2 M KOH electrolyte: (**a**) comparative CV curves at 10 mV/s, (**b**–**e**) CV curves at different scan rates (10 to 80 mV/s), (**f**) peak current versus square root of scan rate, (**g**) diffusion coefficient comparison, and (**h**) log(i) versus log(v) plots for *b*-value determination.

The CV profiles of the PEG-regulated NiWO_4_ electrodes provide clear insight into the profound influence of polymer-assisted interfacial modulation on the electrochemical charge-storage characteristics. Figure 5a presents the CV curves recorded at a scan rate of 10 mV/s within the potential window of 0.05–0.5 V versus Ag/AgCl. All electrodes exhibit well-pronounced anodic and cathodic redox peaks, confirming that the charge-storage mechanism is predominantly governed by reversible Faradaic reactions associated with the Ni^2+^/Ni^3+^ redox transition in alkaline electrolyte. The distinct non-rectangular CV features further verify the pseudocapacitive nature of the NiWO_4_ electrodes, where rapid surface-confined redox processes contribute significantly to the overall capacitance behavior [22]. Among the investigated series, the NiWO-P3 electrode synthesized using 0.3 wt% PEG demonstrates an exceptionally enhanced electrochemical response, evidenced by its substantially enlarged integrated CV area and intensified redox peak currents compared with pristine NiWO and other PEG-modified samples. The anodic and cathodic peaks centered approximately at ~0.38 and ~0.18 V indicate highly reversible electrochemical reactions with fast reaction kinetics and reduced polarization effects. The superior electrochemical activity of NiWO-P3 originates from the optimized role of PEG during hydrothermal crystallization, where the polymer chains effectively regulate nucleation dynamics and suppress uncontrolled particle growth. Such controlled growth behavior facilitates the formation of a highly interconnected porous nanoarchitecture with abundant electrochemically active interfaces and enhanced surface accessibility. In contrast, the pristine NiWO electrode suffers from compact particle aggregation and limited active-site exposure, leading to comparatively sluggish ion diffusion and inferior electrochemical utilization. Similarly, the lower PEG concentration in NiWO-P1 provides insufficient steric stabilization during crystal growth, whereas excessive PEG incorporation in NiWO-P5 induces partial surface blocking and restricted crystallite interaction, ultimately hindering efficient electron transport and electrolyte accessibility [35].

To further investigate the rate-dependent electrochemical behavior, CV measurements were systematically conducted at scan rates ranging from 10 to 80 mV/s (Figure 5b–e). All electrodes preserve their characteristic redox features even at elevated scan rates, highlighting excellent electrochemical reversibility and robust structural stability during rapid charging–discharging processes. As the scan rate increases, the anodic peaks gradually shift toward positive potential while cathodic peaks shift toward negative potential due to increased polarization and ion-diffusion resistance at higher current response. Nevertheless, the retention of the overall CV shape demonstrates the rapid reaction kinetics and efficient electron-transfer characteristics of the engineered NiWO_4_ electrodes [18]. Notably, the NiWO-P3 electrode consistently exhibits the highest current response and broadest CV profile throughout the entire scan-rate range, emphasizing its superior rate capability and enhanced electrochemical kinetics. The unique PEG-mediated porous framework effectively increases electroactive surface exposure while simultaneously generating continuous ion-transport channels within the electrode architecture. This structural advantage significantly improves electrode/electrolyte interfacial interaction and facilitates rapid electrolyte infiltration, thereby maximizing the accessibility of active redox centers [36].

To gain deeper insight into the charge-transfer behavior and ion diffusion kinetics of the PEG-engineered NiWO_4_ electrodes, the electrochemical responses obtained at varying scan rates (10–80 mV/s) were systematically analyzed. As presented in Figure 5f, the anodic and cathodic peak currents exhibit an excellent linear dependence on the square root of the scan rate (*v*^1/2^), indicating that the electrochemical charge-storage process is predominantly governed by diffusion-controlled Faradaic reactions. The strong linearity further confirms efficient electrolyte ion transport within the electrode framework and validates the reversible redox nature of the Ni^2+^/Ni^3+^ electrochemical transition [37]. To quantitatively evaluate the ion diffusion characteristics, the apparent diffusion coefficients (*D*) of the pristine and PEG-modified NiWO_4_ electrodes were estimated using the Randles–Sevcik relationship (1) [38]:

(1) $$D=\frac{i_{p}}{2.69\times 10^{5}\times n^{3/2}\times A\times C\times v^{1/2}}$$

where *i_p_* represents the peak current, *n* denotes the number of electrons involved in the electrochemical reaction, *A* corresponds to the electrochemically active surface area, *C* is the concentration of electrolyte ions, and *v* is the scan rate. The calculated diffusion coefficients at a representative scan rate of 10 mV/s are summarized in Table 1 and comparatively illustrated in Figure 5g. Among all electrodes, the NiWO-P3 sample exhibits the highest diffusion coefficient for both anodic (21.26 × 10^−7^ cm^2^/s) and cathodic (10.55 × 10^−7^ cm^2^/s) processes, demonstrating significantly enhanced ion-transport capability and accelerated electrochemical kinetics, which is substantially higher than that of pristine NiWO and other PEG-assisted variants. The systematic variation in diffusion coefficients across the electrode series clearly demonstrates the critical role of PEG concentration in tailoring the microstructural characteristics and electrochemical transport behavior of NiWO_4_.

To further establish the underlying charge-storage kinetics of the NiWO_4_ electrodes, the relationship between peak current and scan rate was systematically investigated using the power-law expression (2) [39]:

(2) $$i={av}^{b}$$

where *i* represents the measured peak current, *v* denotes the scan rate, and the exponent *b* provides valuable insight into the dominant electrochemical charge-storage mechanism. In general, *b* values approaching 0.5 correspond to diffusion-controlled Faradaic behavior, whereas values nearing 1 indicate surface-controlled capacitive processes. The *b*-values were extracted from the slope of the linear fitting plots between *log*(*i*) vs. *log*(*v*) (Figure 5h), and the obtained values for all electrodes are summarized in Table 1. The calculated *b*-values for the pristine and PEG-modified NiWO_4_ electrodes fall within the range of ~0.52–0.62, clearly confirming that the overall charge-storage mechanism is predominantly governed by diffusion-assisted Faradaic reactions. The obtained values strongly suggest that electrolyte ions actively participate in reversible bulk redox processes through ion insertion/extraction within the NiWO_4_ framework. Simultaneously, the slight deviation of the *b*-values toward higher values indicates the coexistence of surface-controlled pseudocapacitive contributions arising from enhanced electrode/electrolyte interfacial interaction and rapid surface redox activity [40]. The NiWO-P3 electrode exhibits comparatively closer to 0.5 *b*-values relative to the other samples, indicating a more favorable balance between diffusion-driven and surface-controlled electrochemical processes. The synergistic coexistence of these charge-storage mechanisms contributes significantly to the superior electrochemical kinetics observed for the optimized electrode.

Furthermore, to distinguish the individual contributions from capacitive and diffusion-controlled processes, the current response was quantitatively separated according to the following relationship (3) [41]:

(3) $$i\left(V\right)=k_{1}v{+k_{2}v}^{1/2}$$

where the term *k*_1_*v* is related to the surface-controlled capacitive contribution associated with rapid interfacial charge accumulation, while *k*_2_*v*^1/2^ represents the diffusion-governed contribution originating from bulk ion diffusion and Faradaic redox reactions. The constants *k*_1_ and *k*_2_ were determined through linear fitting of *i*(*V*)/*v*^1/2^ versus *v*^1/2^, enabling accurate deconvolution of the total stored charge into capacitive and diffusion-controlled components.

**Figure 6:**
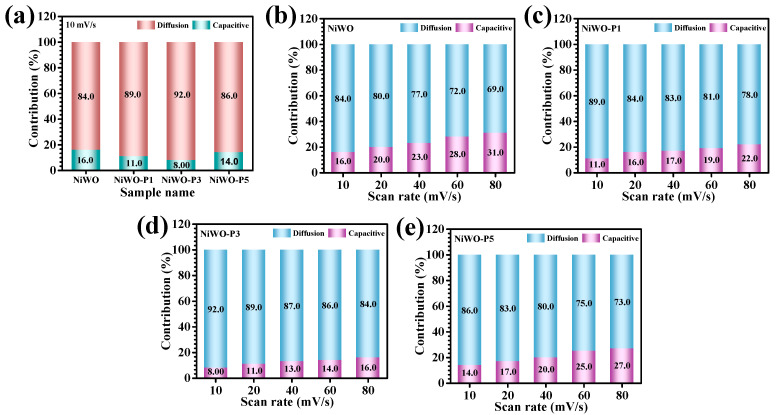
Charge-storage kinetics analysis in a three-electrode configuration using 2 M KOH electrolyte: (**a**) capacitive and diffusion-controlled contribution at 10 mV/s and (**b**–**e**) corresponding contribution profiles at different scan rates (10 to 80 mV/s).

The total stored charge can therefore be expressed as (4) [41]:

(4) $$Q_{t}=Q_{s}+Q_{d}$$

where *Q_s_* and *Q_d_* represent the capacitive and diffusion-controlled charge contributions, respectively. The quantitative analysis performed at a scan rate of 10 mV/s reveals that diffusion-controlled processes dominate the overall charge-storage behavior for all investigated electrodes (Figure 6a). Importantly, the NiWO-P3 electrode delivers the highest diffusion-controlled contribution of 89%, indicating that bulk Faradaic reactions serve as the primary energy-storage mechanism within the optimized electrode system. This dominant diffusion contribution reflects highly efficient electrolyte penetration and enhanced utilization of electrochemically active material throughout the interconnected NiWO_4_ framework. The exceptional diffusion-assisted charge storage observed for NiWO-P3 can be directly correlated with its PEG-regulated porous nanoarchitecture. The optimized structural configuration facilitates rapid OH^−^ ion transport, enlarged electrode/electrolyte contact area, and shortened diffusion pathways, thereby allowing a significantly larger fraction of the electrode volume to actively participate in reversible redox reactions. In comparison, the pristine NiWO electrode exhibits relatively lower diffusion contribution due to dense particle agglomeration and restricted ion accessibility. Similarly, insufficient PEG concentration in NiWO-P1 leads to incomplete structural evolution, whereas excessive PEG incorporation in NiWO-P5 partially obstructs ion diffusion through excessive surface coverage and inhibited crystallite connectivity. Furthermore, the evolution of the charge-storage mechanism with increasing scan rate was systematically analyzed (Figure 6b–e). As the scan rate increases, a gradual enhancement in capacitive contribution is observed for all electrodes. This behavior arises because electrolyte ions possess insufficient time to deeply diffuse into the bulk electrode structure at higher charging rates, thereby shifting the electrochemical response toward surface-dominated reactions. Consequently, fast interfacial redox processes become increasingly prominent under rapid scan conditions [42]. Despite this transition, the NiWO-P3 electrode consistently maintains a substantially higher diffusion-controlled contribution throughout the entire scan-rate range, highlighting its superior ion-transport kinetics and highly optimized structural characteristics.

**Figure 7:**
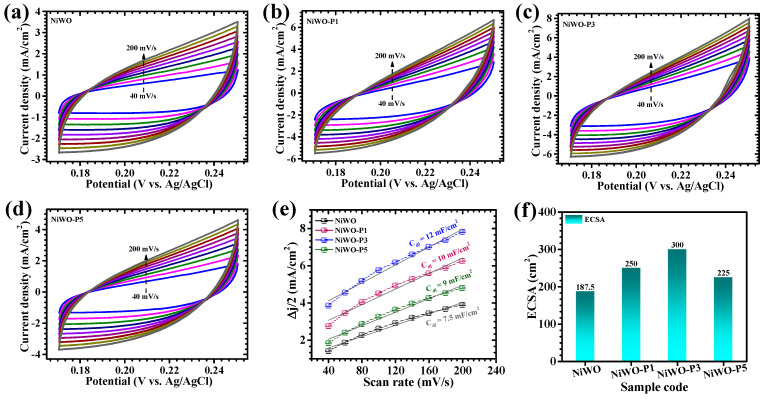
(**a**–**d**) Cyclic voltammetry curves recorded within the non-faradaic potential region in a three-electrode configuration using 2 M KOH electrolyte at different scan rates (40 to 200 mV/s) for all electrodes, respectively. (**e**) Linear fitting plots used for determining the double-layer capacitance (Cdl) values. (**f**) Comparative electrochemical active surface area (ECSA) values.

To further elucidate the influence of PEG-mediated structural modulation on electrochemically accessible active sites, the electrochemical active surface area (ECSA) of the pristine and PEG-engineered NiWO4 electrodes was systematically evaluated through double-layer capacitance (C_dl_) analysis. CV measurements were performed at different scan rates within a carefully selected non-faradaic potential region, where the measured current response predominantly originates from electrochemical double-layer charging without significant contribution from Faradaic redox reactions (Figure 7a–d). The corresponding capacitive current densities extracted at different scan rates were subsequently utilized to determine the C_dl_ values from the linear fitting plots (Figure 7e). The ECSA values were calculated according to Equation (5) [3]:

(5) $$ECSA=\frac{c_{dl}}{C_{s}}$$

where C_dl_ represents the experimentally determined double-layer capacitance and Cs corresponds to the specific capacitance of a flat surface in alkaline electrolyte (0.04 mF/cm^2^). The calculated ECSA values for pristine NiWO, NiWO-P1, NiWO-P3, and NiWO-P5 were determined to be approximately 187.5, 225, 300, and 250 cm^2^, respectively (Figure 7f). Among all investigated electrodes, the optimized NiWO-P3 sample exhibits the highest ECSA value, clearly indicating the presence of substantially enlarged electrochemically accessible surface area and abundant exposed active sites. The significantly enhanced ECSA of the NiWO-P3 electrode can be directly associated with the optimized PEG-assisted nanoarchitectural evolution, which promotes the formation of porous assemblies with improved electrolyte permeability and shortened ion-diffusion pathways. Such structural characteristics facilitate efficient electrolyte penetration and maximize active-site accessibility during electrochemical operation. In contrast, the pristine NiWO electrode exhibits comparatively lower ECSA due to particle agglomeration and limited surface exposure, while excessive PEG incorporation in NiWO-P5 partially suppresses effective active-site accessibility through excessive surface coverage and structural densification. The obtained ECSA results strongly correlate with the observed electrochemical performance trends, further confirming that the optimized PEG concentration plays a critical role in regulating surface accessibility, charge-transfer kinetics, and overall pseudocapacitive charge-storage behavior of the NiWO4 electrodes.

**Figure 8:**
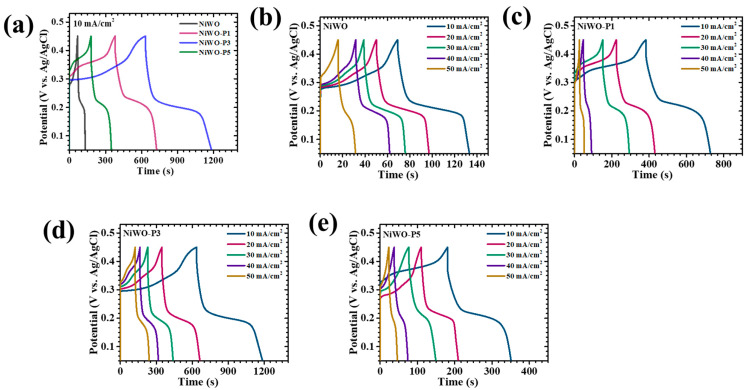
GCD performance of NiWO electrodes in a three-electrode configuration using a 2 M KOH electrolyte: (**a**) comparative GCD curves at 10 mA/cm^2^ and (**b**–**e**) GCD curves at different current densities (10 to 50 mA/cm^2^).

**Figure 9:**
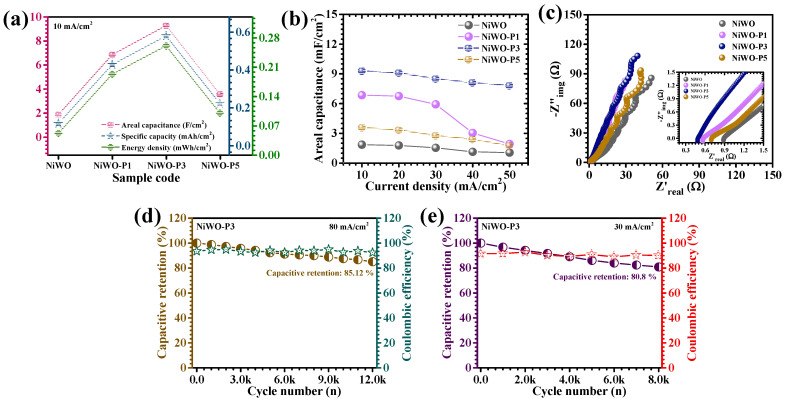
Electrochemical evaluation of NiWO electrodes: (**a**) areal capacitance, (**b**) capacitance retention, (**c**) Nyquist plots, and (**d**) cycling stability of NiWO-P3 over 12,000 cycles at 80 mA/cm^2^ current density, and (**e**) cycling stability of NiWO-P3 over 8000 cycles at 30 mA/cm^2^ current density.

The GCD characteristics of pristine and PEG-tailored NiWO_4_ electrodes were comprehensively examined to gain deeper insight into the role of interfacial nanoengineering on electrochemical energy-storage behavior. Figure 8a displays the comparative GCD curves measured at a current density of 10 mA/cm^2^ within a potential range of 0.05–0.45 V versus Ag/AgCl, whereas the corresponding rate-dependent GCD responses collected between 10 and 50 mA/cm^2^ for all electrodes are presented in Figure 8b–e. Each electrode exhibits distinctly non-triangular charge–discharge characteristics accompanied by noticeable voltage plateaus, which clearly signifies the battery-type pseudocapacitive nature of the NiWO_4_ system governed predominantly by diffusion-controlled Faradaic processes. The departure from ideal linearity mainly arises from reversible redox reactions associated with the Ni^2+^/Ni^3+^ transition occurring within the tungstate lattice framework [22]. Among the investigated samples, the NiWO-P3 electrode demonstrates the longest discharge duration together with highly symmetric charging/discharging behavior, indicating superior electrochemical reversibility and enhanced charge-storage efficiency. The prolonged discharge profile of NiWO-P3 suggests rapid ion-transport dynamics along with highly effective participation of electrochemically active sites throughout the electrode structure. In addition, the relatively slow voltage decay observed for this electrode reflects minimized polarization losses and highly reversible redox activity during the electrochemical process [43]. By comparison, the pristine NiWO electrode delivers a considerably shorter discharge duration and weaker electrochemical performance due to severe particle agglomeration and limited accessibility of active sites. Likewise, the lower PEG content used for NiWO-P1 does not provide sufficient structural modulation, resulting in incomplete morphological optimization and restricted ion diffusion pathways. In contrast, excessive PEG incorporation in NiWO-P5 causes partial surface blocking through excessive encapsulation and viscosity-induced crystallite confinement, which consequently limits electrolyte penetration and charge-transfer efficiency. Such structural drawbacks ultimately reduce electrochemical utilization and suppress the overall capacitive response [44,45]. To quantitatively determine the electrochemical storage capability, the areal capacitance, energy density, and power density were calculated from the nonlinear discharge profiles using the integrated discharge method according to Equations (6)–(8) [11]:

(6) $$C_{A}=\frac{I\times 2\times \int V\left(t\right)dt}{A\times {\left(dV\right)}^{2}}$$

(7) $$ED=\frac{1}{2\times 3600}\;C_{A}\times {dV}^{2}$$

(8) $$PD=\frac{ED\times 3600}{dt}$$

where *I* denotes the discharge current, *∫V*(*t*)*dt* represents the integrated discharge area, *A* corresponds to the effective electrode area, *dV* indicates the operating voltage window, and dt refers to the discharge time. This integrated calculation approach is particularly suitable for pseudocapacitive systems exhibiting nonlinear Faradaic charge–discharge characteristics. At a current density of 10 mA/cm^2^, the areal capacitance values were estimated to be 1.857 F/cm^2^ for pristine NiWO, 6.854 F/cm^2^ for NiWO-P1, 9.284 F/cm^2^ for NiWO-P3, and 3.556 F/cm^2^ for NiWO-P5 (Table 2, Figure 9a). The remarkable capacitance enhancement achieved for NiWO-P3 confirms the critical role of optimized PEG concentration in improving electrochemically accessible surface area and accelerating reaction kinetics. The exceptional electrochemical performance of NiWO-P3 originates from several synergistic contributions: (i) increased active surface exposure, (ii) improved electrolyte-ion transport facilitated by interconnected porous channels, (iii) enhanced electronic conduction through structurally interconnected crystallite networks, and (iv) efficient charge transfer at the electrode/electrolyte interface [29,46]. Although all electrodes display a gradual reduction in capacitance at higher current densities due to insufficient electrolyte penetration under rapid charging conditions, NiWO-P3 retains nearly 84% of its initial capacitance even at 50 mA/cm^2^, highlighting its excellent rate capability and robust structural stability (Figure 9b). These findings clearly demonstrate that optimized PEG-assisted nanoarchitectural engineering significantly promotes ion diffusion, minimizes resistive losses, and enhances reversible Faradaic reactions within the NiWO_4_ framework, thereby making NiWO-P3 a highly promising electrode material for high-performance supercapacitor systems.

EIS measurements were further carried out to investigate the interfacial charge-transfer characteristics and ion diffusion behavior of the NiWO_4_ electrodes. The Nyquist plots recorded over the frequency range of 10 kHz to 0.1 Hz in a 2 M KOH electrolyte (Figure 9c) consist of a compressed semicircle in the high-frequency region followed by an inclined straight line at lower frequencies, corresponding respectively to the charge-transfer process and electrolyte-ion diffusion behavior. The intercept at the high-frequency region along the real impedance axis (Z′) represents the ESR, which includes contributions from electrolyte resistance, intrinsic electrode resistance, and interfacial contact resistance. Meanwhile, the diameter of the semicircle is directly associated with charge-transfer resistance related to Faradaic reaction kinetics at the electrode/electrolyte interface [47]. Significant differences in impedance behavior are observed among the investigated samples, confirming the pronounced influence of PEG-mediated structural tuning on electrochemical transport properties. Notably, the optimized NiWO-P3 electrode exhibits the smallest semicircle diameter together with the steepest low-frequency slope, indicating highly accelerated charge-transfer kinetics and superior ion diffusion efficiency. The extracted ESR values for pristine NiWO, NiWO-P1, NiWO-P3, and NiWO-P5 are summarized in Table 1. The significantly reduced resistance observed for NiWO-P3 confirms its enhanced electrical conductivity and minimized internal resistance losses. Such improved electrochemical characteristics are mainly attributed to the optimized PEG-assisted porous nanoarchitecture, which provides continuous electron-transfer pathways, enlarged electrode/electrolyte interaction area, and efficient ion-transport channels. Conversely, pristine NiWO exhibits comparatively larger impedance owing to strong particle aggregation and poor active-site accessibility. Although NiWO-P1 demonstrates moderate improvement due to partial structural regulation, the insufficient PEG concentration limits complete morphological development. On the other hand, excessive PEG incorporation in NiWO-P5 results in partial surface encapsulation and reduced crystallite interconnectivity, thereby hindering efficient ion transport and interfacial charge transfer.

The long-term electrochemical durability of the optimized electrode was further assessed through continuous cycling measurements for the NiWO-P3 electrode over 12,000 GCD cycles at a high current density of 80 mA/cm^2^, and the corresponding capacitance retention and coulombic efficiency profiles are shown in Figure 9d. Impressively, the electrode preserves nearly 85.12% of its initial capacitance after prolonged cycling, corresponding to only ~15% performance degradation. Such excellent retention behavior demonstrates the remarkable structural integrity and highly reversible Faradaic charge-storage capability of the PEG-engineered NiWO_4_ electrode under rigorous electrochemical operating conditions. The superior cycling stability of NiWO-P3 mainly originates from its optimized porous architecture induced by PEG mediation. The interconnected framework effectively buffers the volume expansion and mechanical stress generated during repeated ion insertion/extraction processes, thereby maintaining structural stability throughout prolonged cycling. Simultaneously, the porous structure promotes continuous electrolyte infiltration and rapid OH^−^ ion transport within the electrode matrix, ensuring sustained active-site accessibility and stable electrochemical kinetics during long-term operation [48]. The slight decrease in capacitance observed after extended cycling may arise from gradual ion trapping and partial accumulation of electrolyte species within microporous regions of the electrode structure. Such localized ion immobilization can subtly reduce the reversibility of Faradaic reactions and limit accessibility to certain electroactive sites during repeated cycling. Nevertheless, the relatively small capacitance decay confirms the excellent electrochemical robustness and structural resilience of the optimized NiWO-P3 electrode [49].

To further validate the electrochemical durability of the optimized NiWO-P3 electrode under comparatively moderate operating conditions, additional cycling stability measurements were carried out at a current density of 30 mA/cm^2^ for 8000 continuous GCD cycles, and the corresponding capacitance retention and coulombic efficiency profiles are presented in Figure 9e. The electrode retains approximately 80.8% of its initial capacitance after prolonged cycling while maintaining stable coulombic efficiency (90%) throughout the cycling process, confirming the excellent reversibility and structural robustness of the PEG-engineered NiWO4 nanoarchitecture under sustained electrochemical operation. The direct in situ growth of NiWO_4_ on conductive nickel foam provides strong interfacial adhesion and minimizes electrode delamination or active-material detachment, thereby contributing significantly to long-term electrochemical stability at such a lower current density. The obtained durability performance under moderate current-density conditions therefore further confirms the practical applicability and robust electrochemical reliability of the NiWO-P3 electrode for advanced high-performance supercapacitor applications.

To further verify the structural robustness of the optimized NiWO-P3 electrode under prolonged electrochemical operation, post-cycling FESEM and EIS analyses were conducted after extended GCD cycling tests. The post-stability FESEM images (Figure S1a,b) reveal that the electrode largely preserves its interconnected porous nanoarchitecture even after long-term cycling, confirming the excellent mechanical and structural stability of the PEG-engineered NiWO_4_ framework. Although slight surface densification and partial agglomeration can be observed after repeated charge–discharge processes, the overall porous morphology and interconnected transport pathways remain effectively intact. This structural preservation is highly beneficial for maintaining continuous electrolyte penetration and efficient ion diffusion during prolonged electrochemical operation. Furthermore, the EIS spectra recorded before and after cycling (Figure S1c) demonstrate only a marginal increase in impedance following stability testing, indicating minimal deterioration in charge-transfer kinetics and interfacial conductivity. The slight increase in ESR (0.66 Ω) after prolonged cycling may originate from gradual ion trapping, partial blockage of active sites, and localized structural stress generated during repetitive OH^−^ ion insertion/extraction processes. Nevertheless, the Nyquist profile after cycling still maintains a relatively low resistance response together with favorable ion-diffusion characteristics, confirming the excellent electrochemical reversibility and durability of the optimized electrode. These findings collectively validate the outstanding structural resilience and long-term electrochemical stability of the NiWO-P3 electrode for advanced supercapacitor applications.

## 5. Electrochemical Performance of Asymmetric Supercapacitor Device

**Figure 10:**
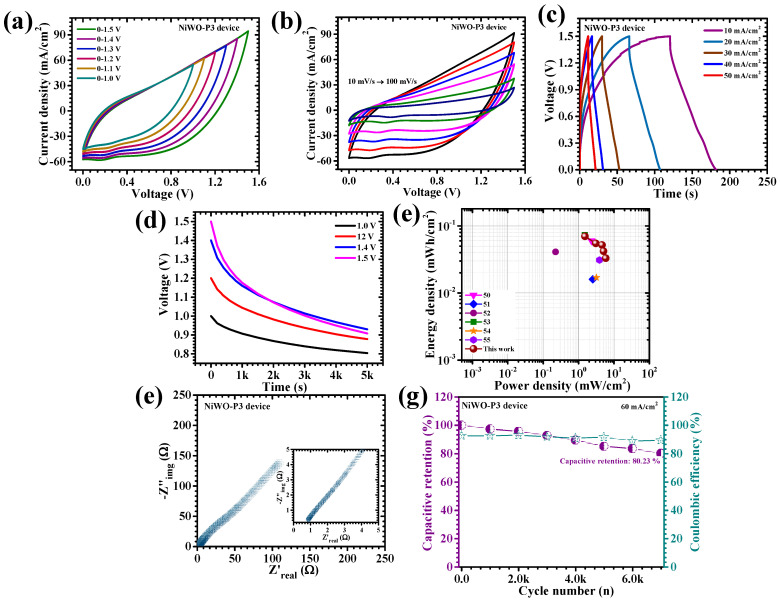
Electrochemical performance of the NiWO-P3//AC asymmetric supercapacitor in a two-electrode configuration using 2 M KOH electrolyte: (**a**) CV curves at different voltage windows, (**b**) CV curves at various scan rates (10 to 100 mV/s), (**c**) GCD curves at different current densities (10 to 50 mA/cm^2^), (**d**) self-discharge behavior of the ASD, (**e**) Ragone plot, (**f**) Nyquist plot, and (**g**) cycling stability over 7000 cycles at 60 mA/cm^2^ current density.

To further assess the practical applicability of the optimized PEG-engineered NiWO_4_ electrode for energy-storage systems, an asymmetric supercapacitor device (ASD) was successfully fabricated using NiWO-P3 as the positive electrode (1 × 1 cm^2^) and the commercially available activated carbon (AC; Thermo Fisher Scientific, Waltham, MA, USA; 100% compressed, Lot: Y23H004) as the negative electrode (1 × 1 cm^2^). This asymmetric configuration was intentionally designed to integrate the diffusion-controlled Faradaic charge-storage behavior of NiWO_4_ with the rapid electric double-layer capacitive characteristics of AC, thereby enabling simultaneous enhancement of energy density and power performance. Both electrodes were deposited on conductive nickel foam substrates, while a 2 M KOH electrolyte and a porous separator (filter paper) were employed to facilitate efficient ion transport and stable electrochemical operation. The electrochemical properties of the assembled NiWO-P3//AC device were systematically analyzed through CV, GCD, and EIS measurements in a two-electrode configuration. Initially, the optimum operating voltage window of the asymmetric device was established through CV analysis recorded over progressively expanded potential ranges from 0–1.0 V to 0–1.5 V (Figure 10a). As the voltage range increased, the enclosed CV area gradually expanded without obvious distortion of the curve shape or sudden current fluctuations, indicating excellent electrochemical reversibility and stable device operation. Importantly, the device maintained stable electrochemical characteristics up to 1.5 V, confirming negligible polarization effects and the absence of significant electrolyte decomposition within the optimized potential window. Figure 10b presents the CV curves of the NiWO-P3//AC device measured at scan rates between 10 and 100 mV/s within the optimized voltage range of 0–1.5 V. The CV profiles display a hybrid characteristic combining quasi-rectangular behavior with noticeable redox peaks, indicating the synergistic coexistence of electric double-layer capacitance contributed by AC and reversible Faradaic reactions originating from the NiWO_4_ electrode. Moreover, the overall CV shape remains highly stable even at higher scan rates, demonstrating excellent electrochemical compatibility between the positive and negative electrodes together with rapid ion/electron transport kinetics.

The GCD curves recorded at different current densities ranging from 10 to 50 mA/cm^2^ are shown in Figure 10c. The nonlinear charge–discharge profiles accompanied by subtle voltage plateaus further verify the dominant pseudocapacitive behavior of the asymmetric device. At a current density of 10 mA/cm^2^, the NiWO-P3//AC ASD delivers a high areal capacitance of approximately 224 mF/cm^2^ along with an energy density of 0.07 mWh/cm^2^ and a power density of 1.52 mW/cm^2^ (Table 3).

The enhanced electrochemical performance originates from the synergistic integration of the highly redox-active porous NiWO_4_ framework with the conductive AC electrode, which together promote rapid electrolyte-ion diffusion, efficient charge-transfer kinetics, and improved electrochemical utilization during repeated charging/discharging operation. The self-discharge profiles (Figure 10d) reveal an initial rapid voltage decay followed by comparatively slower relaxation at longer durations, which is characteristic of pseudocapacitive aqueous supercapacitor systems. The faster voltage decay observed at higher operating voltages can be attributed to enhanced polarization effects and increased parasitic side reactions under elevated charging conditions. Nevertheless, the device preserves appreciable voltage retention even at 1.5 V, indicating stable charge-storage reversibility and favorable electrochemical reliability of the optimized NiWO-P3//AC asymmetric configuration.

The Ragone plot derived from the two-electrode GCD measurements demonstrates the excellent energy–power characteristics of the NiWO-P3//AC asymmetric supercapacitor device (Figure 10e) [50,51,52,53,54,55]. The device delivers a maximum energy density of 0.07 mWh/cm^2^ at a power density of 1.52 mW/cm^2^ and retains 0.033 mWh/cm^2^ even at a higher power density of 5.88 mW/cm^2^. Furthermore, the obtained electrochemical performance compares favorably with several previously reported related supercapacitor systems, highlighting the effectiveness of the optimized PEG-engineered NiWO_4_ nanoarchitecture for high-performance energy-storage applications. To gain further understanding of the interfacial electrochemical behavior, EIS measurements were conducted for the assembled asymmetric device. As illustrated in Figure 10f, the Nyquist plot displays a relatively small semicircle in the high-frequency region followed by a steep low-frequency line, indicating low charge-transfer resistance and efficient ion diffusion within the device architecture. The ESR value of the NiWO-P3//AC device was calculated to be approximately 0.86 Ω, reflecting excellent electrical conductivity and highly favorable electrode/electrolyte interfacial contact. In addition, the long-term electrochemical durability of the NiWO-P3//AC device was examined through continuous GCD cycling at a current density of 60 mA/cm^2^ for 7000 cycles (Figure 10g). Impressively, the device retains nearly 80.23% of its initial capacitance while maintaining high coulombic efficiency after prolonged cycling. The excellent cycling stability confirms the remarkable structural durability and highly reversible electrochemical behavior of the optimized NiWO_4_ electrode during repeated ion insertion/extraction processes. The PEG-assisted porous structure effectively accommodates volume fluctuations during continuous cycling while preserving structural integrity and electroactive-site accessibility throughout prolonged electrochemical operation. Overall, the outstanding electrochemical properties demonstrated by the NiWO-P3//AC ASD, including its wide operating voltage range, high areal capacitance, low internal resistance, excellent rate capability, and superior cycling stability, clearly validate the effectiveness of PEG-assisted nanoarchitectural engineering in optimizing the electrochemical functionality of NiWO_4_ electrodes for advanced high-performance energy-storage applications.

The comparative benchmarking analysis presented in Table 4 [56,57,58,59,60,61] clearly demonstrates that the optimized NiWO-P3//AC asymmetric supercapacitor device exhibits highly competitive electrochemical performance relative to previously reported related energy-storage systems. The device delivers an excellent balance between energy density and power density while simultaneously maintaining remarkable long-term cycling durability. Such enhanced electrochemical characteristics can be directly attributed to the optimized PEG-mediated porous nanoarchitecture, which promotes enlarged electrochemically active surface area, accelerated electrolyte-ion diffusion, and improved charge-transfer kinetics. The interconnected electrode framework further facilitates efficient utilization of redox-active sites and minimizes resistive losses during repeated charge–discharge operation. These results collectively highlight the significant potential of the engineered NiWO_4_ electrode system for advanced high-performance supercapacitor applications.

## 6. Conclusions

In conclusion, PEG-assisted interfacial modulation was successfully employed to regulate the structural and electrochemical characteristics of hydrothermally synthesized NiWO_4_ electrodes. The findings reveal that PEG concentration strongly influences crystal growth, morphology evolution, and electrochemical accessibility. Among all samples, the NiWO-P3 electrode synthesized with 0.3 wt% PEG developed a highly interconnected porous nanograin architecture, providing improved electrolyte penetration and accelerated ion transport pathways. As a result, NiWO-P3 delivered the highest areal capacitance of 9.284 F/cm^2^ at 10 mA/cm^2^ with nearly 84% capacitance retention at higher current density. The optimized electrode also exhibited enhanced diffusion kinetics and retained 85.12% capacitance after 12,000 cycles, demonstrating excellent structural stability and reversible electrochemical behavior. Furthermore, the NiWO-P3//AC asymmetric device displayed promising electrochemical performance with good cycling durability, confirming its practical applicability for energy-storage systems.

## Figures and Tables

**Table 1:** Summary of kinetic and electrochemical parameters, including diffusion coefficients, *b*-values, and equivalent series resistance (ESR) of the investigated electrodes.

Sample Code	Diffusion Coefficient (cm^2^/s) × 10^−7^		*b*-Value	ESR (Ω)
	Oxidation	Reduction		
NiWO	6.844	2.7633	0.62	0.88
NiWO-P1	15.992	7.194	0.59	0.56
NiWO-P3	21.26	10.549	0.52	0.48
NiWO-P5	9.51	4.687	0.6	0.7

**Table 2:** Electrochemical performance evaluation of the electrodes, presenting areal capacitance, areal capacity, energy density, and power density at different current densities.

Sample Code	I (mA/cm^2^)	Areal Capacitance C_A_ (F/cm^2^)	Capacity (mAh/cm^2^)	Energy Density ED (mWh/cm^2^)	Power Density PD (mW/cm^2^)
**NiWO**	10	1.857	0.116	0.052	1.67
	20	1.778	0.111	0.050	3.32
	30	1.541	0.096	0.043	4.86
	40	1.136	0.071	0.032	6.27
	50	1.057	0.066	0.030	7.19
**NiWO-P1**	10	6.854	0.428	0.193	1.73
	20	6.756	0.422	0.190	3.35
	30	5.926	0.370	0.167	4.79
	40	3.042	0.190	0.086	6.92
	50	1.926	0.120	0.054	7.68
**NiWO-P3**	10	9.284	0.580	0.261	1.50
	20	9.096	0.569	0.256	2.97
	30	8.494	0.531	0.239	4.34
	40	8.099	0.506	0.228	5.58
	50	7.802	0.488	0.219	6.75
**NiWO-P5**	10	3.556	0.222	0.100	1.64
	20	3.319	0.207	0.093	3.36
	30	2.746	0.172	0.077	5.00
	40	2.370	0.148	0.067	6.67
	50	1.728	0.108	0.049	7.61

**Table 3:** Electrochemical performance of the NiWO-P3//AC ASD presenting at different current densities.

Sample Code	I (mA)	Areal Capacitance CA (mF/cm^2^)	Capacity (mAh/cm^2^)	Energy Density ED (mWh/cm^2^)	Power Density PD (mW/cm^2^)
NiWO-P3 device	10	224	0.047	0.070	1.52
	20	176	0.037	0.055	3.07
	30	168	0.035	0.052	4.30
	40	135	0.028	0.042	5.07
	50	104	0.022	0.033	5.88

**Table 4:** Comparative electrochemical performance of the NiWO-P3//AC ASD with previously reported supercapacitor systems.

Device Material	Electrolyte	Voltage	Capacitance	Energy/Power Density	Cycle Life	Refs.
Ni-MOF//AC	3 M KOH	1.4 V	87 F/g	21.05 Wh/kg 6.03 kW/kg	70% (2000 cycles)	[56]
ZIF-PPy//ZIF-PPy	1 M Na_2_SO_4_	0.6 V	225.8 mF/cm^2^	0.0113 mWh/cm^2^ 1.44 mW/cm^2^	-	[57]
CoNi_2_S_4_//(YS-CS)	2 M KOH	1.5 V	98 F/g	35 Wh/kg 5.760 kW/kg	63.18% (5000 cycles)	[58]
CC/CoNi//g-CNT	1 M KOH	1.5 V	846 mF/cm^2^	55.5 Wh/kg 175.5 W/kg	96.5% (10,000 cycles)	[59]
MoS_2_-ZIF//ZDPC	1 M LiPF_6_	4.0 V	-	155 Wh/kg 20 kW/kg	99.9% (10,000 cycles)	[60]
PET/MOF-1/rGO/PPy//PET/ MOF-1/rGO/PPy	PVA + H_2_SO_4_ gel	1.0 V	150 mF/cm^2^	64 μWh/cm^2^ 0.03 mW/cm^2^	85% (1000 cycles)	[61]
NiWO-P3//AC	2 M KOH	1.5 V	224 mF/cm^2^	0.07 mWh/cm^2^ 1.52 mW/cm^2^	80.23% (7000 cycles)	This work

## Data Availability

The data presented in this study are available on request from the corresponding author due to privacy reasons.

## Supplementary Materials

The following supporting information can be downloaded at: https://www.mdpi.com/article/10.3390/polym18131639/s1, Figure S1: (a,b) After stability FESEM images of the NiWO-P3 electrode, (c) Before and after stability EIS.
