# Supplementary material for: Interfacial Modulation of Nickel Tungstate by Polyethylene Glycol Toward Enhanced Electrochemical Energy Storage
Source: Polymers (Basel). 2026 Jul 1;18(13):1639. doi: 10.3390/polym18131639 (PMC13364022; doi:10.3390/polym18131639)
Supplement: Supplementary file 1 [file polymers-18-01639-s001.zip › polymers-4378454-supplementary.pdf]

Interfacial Modulation of Nickel Tungstate by Polyethylene Glycol toward Enhanced Electrochemical Energy Storage

Chaitany Jayprakash Raorane *, Seong- Cheol Kim

School of Chemical Engineering, Yeungnam University, Gyeongsan, Gyeongsanbuk-Do 38541, Republic of Korea

* Correspondence: Chaitany Jayprakash Raorane (chaitanyaraorane22@ynu.ac.kr)

Supplementary information

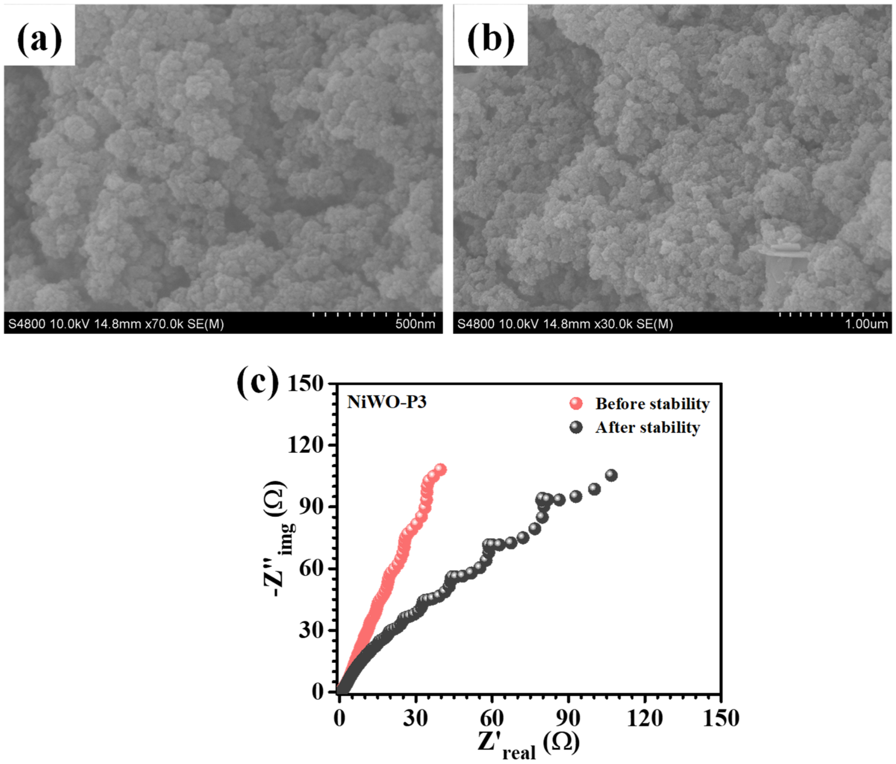

Fig. S1: (a,b) After stability FESEM images of the NiWO-P3 electrode, (c) Before and after stability EIS.
